# Is the lag screw sliding effective in the intramedullary nailing in A1 and A2 AO-OTA intertrochanteric fractures? A prospective study of Sliding and None-sliding lag screw in Gamma-III nail

**DOI:** 10.1186/1757-7241-20-60

**Published:** 2012-09-01

**Authors:** Yi Zhu, Severin Meili, Changqing Zhang, Congfeng Luo, Bing-fang Zeng

**Affiliations:** 1 Department of Orthopaedics, Shanghai Sixth People’s Hospital, Shanghai Jiaotong University, No.600, YiShan Road, Xuhui District, Shanghai, 200233, China; 2 Fellow of Trauma, Kantonsspital Winterthur, Winterthur, Switzerland

**Keywords:** Intertrochanteric fractures, A1 and A2 AO-OTA, Gamma nail, Lag screw sliding

## Abstract

**Object:**

To compare the Sliding with Non-sliding lag screw of a gamma nail in the treatment of A1 and A2 AO-OTA intertrochanteric fractures.

**Materials and methods:**

80 patients were prospectively collected. In each group, AO/OTA 31-A were classified into group A. AO/OTA 31-A2.1 was classified as group B. We classified the A2.2 and A2.3 as group C. According to the set-screw locking formation of Gamma-III, the cases were randomly allocated to Sliding subgroup and Non-sliding subgroup in A, B and C groups. Follow-ups were performed 1, 3, 6 and 12 months postoperatively.

**Results:**

In the Sliding group, the bone healing rate 3, 6, 12 months postoperatively reached 85.00%, 97.50%, 100% in group A, B and C. Meanwhile, in Non-sliding group, postoperatively, bone healing rate were 90.00%, 95.00% and 97.50% in group A, B and C, respectively. Both differences were not significant. Lower limb discrepancy between Sliding and Non-sliding pattern was significantly different in group C which represent fracture types of AO/OTA 31-A2.2 and A2.3 (0.573 ± 0.019 mm in Non-sliding group, 0.955 mm ± 0.024 mm in Sliding group, P < 0.001 ). Difference of sliding distance among the three groups was significant among group A, B and C: 0.48 mm ± 0.04 mm, 0.62 mm ± 0.07 mm and 0.92 mm ± 0.04 mm (P < 0.001). Differences in average healing time and Harris scores also presented no significance in the three groups.

**Conclusions:**

As a result, we can conclude that the sliding distance is minimal in Gamma nails and it is related to the comminuted extent of the intertrochanteric area in A1 and A2 AO-OTA intertrochanteric fractures. For treating these kinds of fractures, the sliding of the lag screw of an Gamma nail does not improve any clinical results and in certain cases, such as highly comminuted A1 and A2 fractures, can therefore even benefit from a locked lag screw by tightening the set-screw.

## Introduction

The Dynamic Hip Screw (DHS) and the Gamma Nail are the most acceptable fixation methods to treat intertrochanteric fractures [1-5]. The best advantage of the DHS is the dynamical lag screw, which can promote the compression of the fracture line [6-8]. This technique has been proved to stimulate the callus formation [9,10] and applied widely such as dynamic compression plate, external fixation and dynamization of intramedullary nailing [11,12].

The Gamma Nail is a stiffer implant and has a shorter lever arm, which makes itself a load-sharing device. Furthermore, biomechanical experiments supported that the sliding ability of the lag screw was maintained in the Gamma Nail, but decreased in comparison with the DHS device [7,13,14]. Up to the present, no clinical results support whether the Gamma nail can take advantage of the sliding screw, especially in stable intertrochanteric fractures. Our goal is to compare the healing time, Harris Hip Score, lower limb discrepancy as well as the sliding distance between two groups with a sliding and non-sliding lag screw pattern in the Gamma Nail among 31-A1 and A2 AO-OTA intertrochanteric fractures. In order to avoid the impact of osteoporosis on the device [2,15], only younger healthy patients with better bone quality were enrolled in our study.

## Materials and methods

### Patient selection and grouping

From 2008.1.1 to 2010.12.31, a total of 325 cases suffered from intertrochanteric fractures in the clinical center of orthopedic trauma in Shanghai Sixth People’s hospital. The inclusion criteria are: (1) age >18 and < 60 years old, (2) fresh, closed fracture, (3) no combined fracture, (4) AO/OTA 31-A1, A2 with integrate lateral wall. The exclusion criteria are: (1) pathological or open fracture, (2) combined with diseases of the cardiac system, the hematologic system or other systems, (3) osteoporosis (Singh index ≦ 3). Among these, 80 patients satisfied the criteria and were undergone fixation by Gamma-III nail (Stryker, Schonkirchen, Germany).

**Figure 1:**
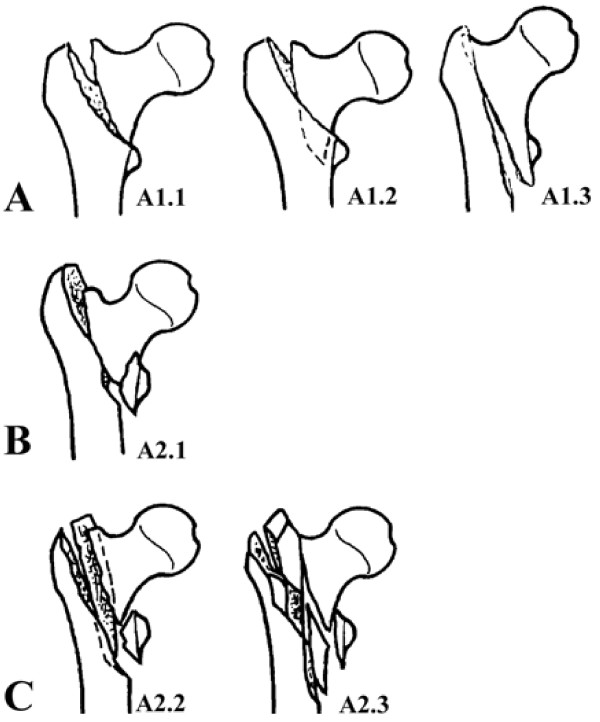
The enrolled fracture types and grouping based on the AO/OTA classification: Group A: AO/OTA 31-A1; Group B: AO/OTA 31-A2.1; Group C: AO/OTA 31-A2.2, 2.3.

All the cases were chosen as 31-A1 and A2 fractures determined by AO/ASIF classification because they are intertrochanteric fractures without the reversed type oblique fracture line. In our study, we classified these cases into group **A, B** and **C** for the differences of their comminution (Figure 1). We classified the A2.2 and A2.3 as group C because they show comminution around the intertrochanteric area and therefore always belong to the unstable patterns [16,17]. All the cases were classified simultaneously by 2 observers, a junior-level and fellowship-trained orthopedic traumatologist (YZ and SM.) and agreement was reached by consensus. Inter-observer reliability measurements were reviewed and reached “substantial agreement”. Intra-observer reliability measurements were not performed.

According to the set-screw locking pattern of Gamma Nail [18], the cases were randomly divided into a sliding subgroup and non-sliding subgroup within groups A, B and C. Sliding or non-sliding is defined by tightening or not tightening the set-screw. Randomization was achieved by drawing an unseen card from a sealed envelope to separate the patients equally into “Sliding group” and “Non-sliding group”.

### Interventions

**Figure 2:**
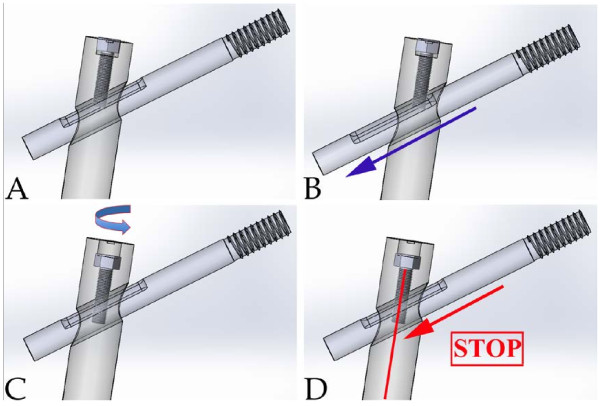
**A schematic view of the lag screw sliding in the Gamma Nail.** If the set screw was not tightened (as showed in **A**), after postoperative load bearing, the sliding of the lag screw (blue arrow) will allow fracture site impaction (**B**). If the set screw is tightened (**C**) no sliding will occur (**D**).

All operations were performed by the same surgeon (CQZ), who had at least 20 years experience in orthopedic trauma. Patients were positioned on the orthopedic traction table and total anesthesia was used. Closed reduction was performed with the aid of C-arm fluoroscopy. Operations were performed adhered to standard protocols for the Gamma-III nail [18]. A Gamma Nail is a 180 mm Titanium Alloy with Type II proximal anodization with distal diameters of 15.5 mm and 11 mm. Reaming of the medullary canal was generally performed for all the cases before insertion. The distal locking screw was used. Tightening or not tightening the set-screw in the procedure was according to the randomized card. A sketch map of a lag screw sliding of Gamma is depicted in Figure 2. Drainage tube was placed for each patient for 1 day. None of them needed transfusion postoperatively. Each patient received regular antibiotic prophylaxis for only 1 day postoperatively. Painkillers were not suggested for the patients. The rehabilitation protocol contained two days of bed rest followed by ambulation with immediate weight bearing, eventually allowing the screw sliding and bone impaction. No other physiotherapy was suggested to the patients.

### Parameter assessment

Radiographic observations assessed postoperatively in X-rays were complications, visibility of the fracture line (respectively callus formation) and radiographic bone healing. The bone healing, defined as the presence of bridging callus and the absence of the fracture line, was assessed as radiological union. Clinical judgment was based upon the subjective impression of fracture site pain and fracture stiffness under physical load. Patients were performed a full-leg radiograph 6-months postoperatively and limb length was estimated in PACS system by drawing a line from the top of the femoral head to the center of the tangential line drawn between the most distal part of the medial and lateral femoral condyle as suggested by Platzer et al. [2]. The differences between the distance of the injured and uninjured side were recorded as length of lower limb discrepancy. The measurements of the femoral length of all radiographs were repeated by a second surgeon, to assess the inter-observer variability. At the same time, we used a special radiographic method suggested by Lunsjo et al. [19] for calculating the distance of the lag screw sliding.

**Figure 3:**
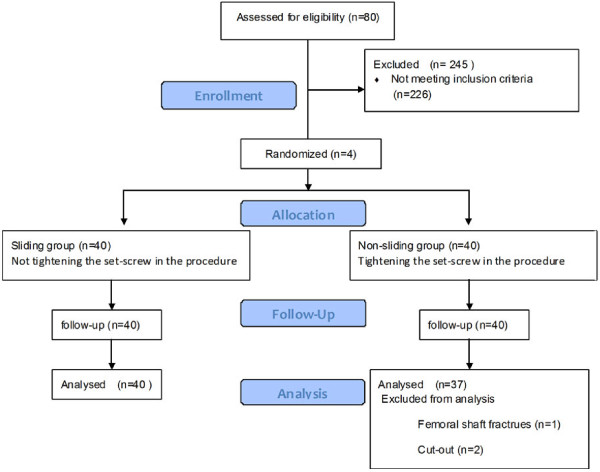
Flow Diagram for our RCT.

Follow-ups were performed 1, 3, 6 and 12 months postoperatively. At each postoperative control, radiographs were made and any change in the position of the implant, complication, or fixation failure was recorded. On the 6-months radiograph, the lag screw sliding distance as well as the femoral length discrepancy were calculated between Sliding and non-sliding groups. All measurements were made three times and the means were finally recorded. The Harris hip score was chosen to assess the hip function of the patients 12 months postoperatively in the outpatient clinic. The statistics were measured by YZ and CQZ and were finally recorded before agreement was reached. All the CONSORT guidelines for our study can be read in Figure 3. The study was approved by the Committee of Medical Ethics and the Institutional Review Board of our University.

### Statistical analyses

The Chi square Test was used to analysis the differences between the basic information of the two groups. Significance was set at p < 0.05.

To analysis the differences of the Harris hip score, lower limb discrepancy and the healing rate between the Sliding group and Non-sliding group, comparison was performed between the both. A measurement of the sliding distance in the sliding group among groups A, B and C was also performed. The Mann–Whitney U test was used to test for independent groups. Significance was set at p < 0.05.

## Results

**Figure 4:**
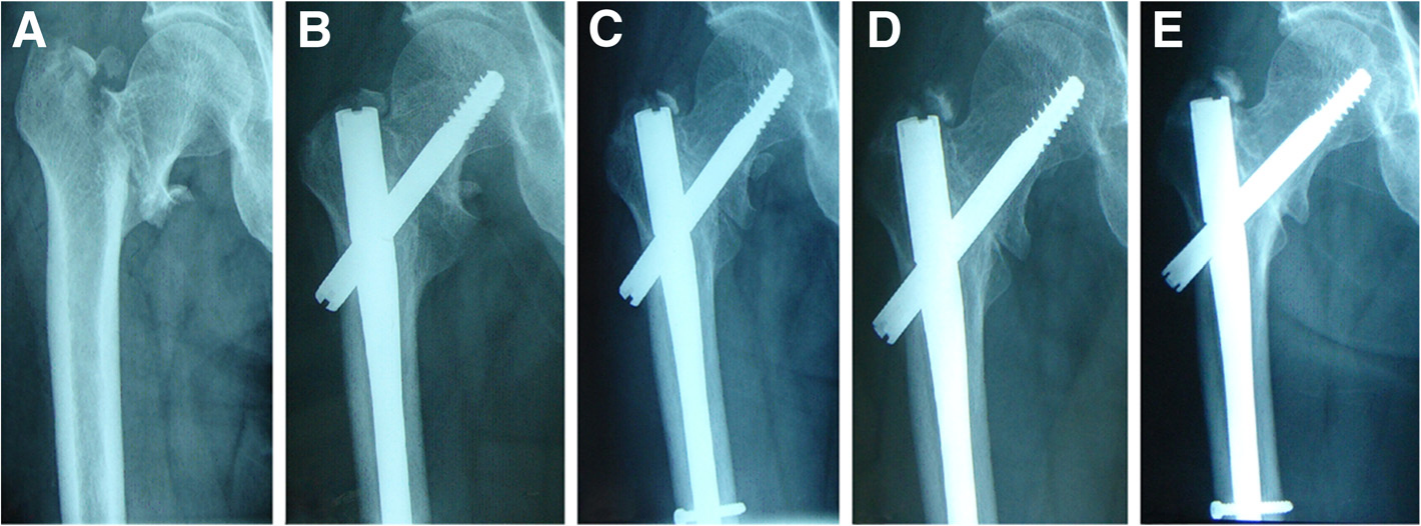
A. 47 years old, male (AO/OTA: 31-A1.2) B.1 month after operation C. 3 months after operation D. 6 months after operation E. 12 months after operation.

All the follow-up information is schematically listed in Table 1. The whole information was verified from the charts in the patient history room. Both groups were well matched; all these differences were not significant (NS), especially the average healing time and the Harris hip score in the three groups. The X-rays of a representative case is shown in Figure 4. In one case a cut out phenomenon of the femoral head was observed. Two cases suffered from femoral shaft fractures.

**Table 1:** Comparison of patients variables between the Sliding group and Non-sliding group

**Variables**	**Sliding group**	**Non-sliding group**	**P values**
Patients(n)	40	40	
Age(y)	45.55 ± 9.87	46.83 ± 8.76	0.453
Gender (Male/Female)	24/16	30/10	0.232
Side (Left/Right)	15/25	19/21	0.498
Weight 1M postoperatively (kg)	64.83 ± 8.79	65.93 ± 8.68	0.575
AO classification			
Group A (31-A1)	7	4	
Group B (31-A2.1)	12	16	0.493
Group C (31-A2.2, A2.3)	21	20	
Operation time (min)	46.73 ± 10.37	48.35 ± 9.23	0.461
Intra-operative blood loss (ml)	141.1 ± 18.12	138.5 ± 19.42	0.538
Tip-Apex Distance (mm)	22.03 ± 4.19	21.23 ± 3.12	0.336
Fracture reduction (mm)	8.48 ± 3.34	8.98 ± 3.50	0.515
Length of hospital stay (days)	4 ± 1	4 ± 1	NA
Bone healing cases (postoperatively)	40	40	
3M	34(85.00%)	36(90.00%)	
6M	39(97.50%)	38(95.00%)	NA
12M	40(100%)	39(97.50%)	
Complications			
Group A (31-A1)	0	0	
Group B (31-A2.1)	0	2 (Femoral shaft fracture)	NA
Group C (31-A2.2, A2.3)	0	1 (Cut-out)	
Average healing time (Months)			
Group A (31-A1)	3.00 ± 1.13	3.00 ± 0.00	1
Group B (31-A2.1)	3.25 ± 0.87	3.25 ± 0.87	
Group C (31-A2.2, A2.3)	3.23 ± 0.81	3.85 ± 3.64	
Harris Hip Score			
Group A (31-A1)	88.00 ± 8.10	90.25 ± 5.12	0.927
Group B (31-A2.1)	86.33 ± 11.85	87.13 ± 6.60	
Group C (31-A2.2, A2.3)	84.52 ± 5.51	85.60 ± 10.34	

**Figure 5:**
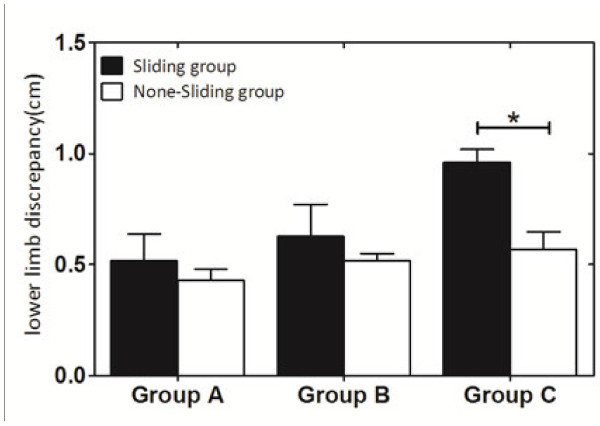
**Lower limb discrepancy between Sliding and Non-sliding groups.** P < 0.001. Mean ± SD, *Significantly different at p < 0.05.

**Figure 6:**
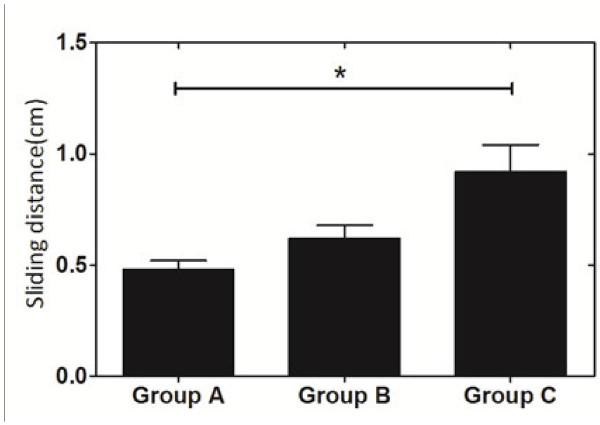
**Sliding distance in the Sliding group among groups A, B and C.** P < 0.001. Mean ± SD. *significantly different at p < 0.05.

Lower limb discrepancy between Sliding and Non-sliding pattern was significantly different in group C which represent fracture types of AO/OTA 31-A2.2 and A2.3 (0.573 mm ± 0.019 mm in Non-sliding group, 0.955 mm ± 0.024 mm in Sliding group, P < 0.001) Figure 5. Difference of sliding distance among the three groups was significant among group A, B and C: 0.48 mm ±0.04 mm, 0.62 mm ± 0.07 mm and 0.92 mm ± 0.04 mm (P < 0.001) Figure 6. Differences in average healing time and Harris scores also presented no significance among group A, B and C.

## Discussion

The similar mechanic characteristic of the Gamma Nail and DHS is the lag screw, which has a mechanical sliding ability of the head-neck fragment relative to the shaft fragment in the intertrochanteric area [7,8,14,20]. Adequate purchase of the proximal screw within the femoral head, sufficient stability of the implant and proper sliding of the head-neck fragment provide better stability in the Gamma Nail [14]. It has been mechanically proved that the sliding ability of the lag screw in the Gamma Nail depends on the angle between the screw and the nail as well as the extent of the engaged screw within the barrel of the device [21,22]. Interestingly, there is a small tip in the manipulation of the Gamma Nail [18] which created the idea of our study. As the technique brochure suggests, after slightly tightening the set screw it could then be unscrewed by one quarter of a turn. This ensures a free sliding of the lag screw, which may stimulate the callus formation when early weight bearing is permitted. In contrary, if the set screw is tightened, the sliding is stopped. Therefore, now obtaining a more rigid fixation, we wanted to investigate the clinical benefit from the sliding screw in the Gamma Nail.

According to the AO/ASIF classification, A1 and A2 intertrochanteric fractures, excluding the reversed type fractures as well as a broken lateral wall, there is a good indication for implanting both, the Gamma Nail or the DHS [17,23]. Therefore, we aimed to choose these types to investigate the sliding effect in Gamma Nails. To avoid the impact of osteoporosis on the device [2,15], only younger healthy patients were enrolled. In the sliding group, we could show that the difference of the sliding distance 6 months postoperatively is significant among group A, B and C, but less than reported in previous studies on the DHS [24]. To the best of our knowledge, a larger bending moment and higher force is required to initiate the sliding in the DHS, due to the longer leverage arm of the implant. Thus, the bending moment is increased in a heavier patient, by a smaller screw-nail angle and by a longer extension of the screw [7,20,21]. Maximum engagement of the screw in the barrel and less screw-plate angle would be greater ease of sliding with increasing bone impaction and stability at the fracture site Maximum engagement of the screw in the barrel and less screw-plate angle hinder the sliding with increased bone impaction and stability at the fracture site [22]). All Gamma Nails preferably have a small nail-screw-angle between the nail and the screw because of operative and design-related constraints, which increases the bending moment. The distance over which the screw is engaged in a Gamma nail is shorter than the distance over which it is engaged in a DHS. This fact decreases the ability of the screw to slide through the intramedullary device. Therefore, the sliding properties of commercially available second-generation intramedullary devices would be expected to be lower than those of smaller-angle DHS. That’s why the sliding distance in Gamma Nails is fairly short. Furthermore, the rehabilitation protocol consisted of 2 days of bed rest followed by ambulation with immediate weight bearing, eventually allowing the screw sliding and bone impaction. To keep the groups well comparable, both were well matched in fracture type, patients’ body weight. Furthermore, our study showed that there is no significance in both groups in the tip-apex-distance (TAD) as well as in the accuracy of fracture reduction. We finally consider that the type of fracture may be one of the reasons, which affects the sliding distance in the Gamma Nail. The more fracture comminution around the intertrochanteric area, the more sliding distance can be observed**.**

One of the well-known complications caused by the sliding of the lag screw is shortening of the leg, which consequently can affect the hip function [6,25]. As proved previously, Osteoporosis, unstable fracture types and early mobilization with immediate weight bearing are also main reasons for lower limb discrepancy [20,24,26]. But no comparative study of the Gamma Nail itself, which is mechanically a quite different apparatus in comparison with the DHS in sliding of the lag screw, has been conducted. So far, only one research group focused on the leg shorting using the Gamma nail. Platzer et al. [2] revealed a mean femoral shortening of 10.6mm after fixation by Gamma Nail in unstable fracture types (31-A2andA3), while measuring a mean femoral inequality of 5.5 mm in the stable fracture types (31-A1). In our study, we also utilize the same fixation and grouped the different fracture patterns in order to limit the impact of the comminution for the lag screw sliding or femoral shorting. Therefore, we consider that the results of our research are more convincing in the ascertainment of the lag screw influence on Gamma Nail. Finally, we find significant differences in lower limb length inequality in group C between the sliding and non-sliding lag screw. However, the difference between group A (AO/OTA 31-A1) and group B (AO/OTA 31-A2.1) is not significant. Because both groups are well matched, we presume that most likely the reason of lower limb discrepancy is the sliding of the lag screw. The fracture type may be the main reason for the sliding of the lag screw in both groups. Platzer et al. [2] thought that the unstable fracture might cause more shorting. Our data proved that the more comminution in the intertrochanteric area (from group A through C), the more sliding of lag screw, and thus a larger discrepancy lower limb length could be observed in the Gamma Nail.

The bone healing rate in our study is high. Nearly all the cases healed in 12 months postoperatively. Interestingly, the bone healing rate and average healing time shows no relation to the sliding characteristic in our study. The occurred complications were femoral shaft fractures (2 cases) and one screw cut-out in the non-sliding group, but healed eventually after additional fixation. The healing rate and number of complications are equal to former studies [3,4]. Excessive interfragmental movement may cause bone stiffness and nonunion [10,27]. Although this is rare in intertrochanteric fractures fixed by Gamma Nail [27,28] and we didn’t observe that problem in our collective. Early operation and early mobilization resulted in a good functional outcome in all patients. In our research, all the patients were instructed to ambulate with partial weight-bearing two weeks after operation in order to get a good rehabilitation result and fracture site impaction. The HHS [29] was used as objective assessment to evaluate the hip function after operation to test whether the lower limb inequality would affect the hip function. To our knowledge, shortening of the lower limb may cause two problems: firstly, the serious limb inequality would lead to a limping gait. Secondly, the adduction strength would decrease with the lower moments. Our research showed no difference of the HHS between each subgroup. We consider both groups to have equivalent results, although shortening of the leg plays little role in the hip function, it is very important to avoid limb length inequality.

The cut-out phenomenon is one of the most common complications of the Gamma Nail. It is often caused by osteoporotic bone and incorrect lag screw positioning [3]. Some biomechanical study even illustrate that impeded sliding of the lag screw may even be one reason of the cut-out phenomenon [8,22]. In our study, one patient suffered from cut out because of incorrect lag screw positioning. We could not find any evidence that non-sliding of the lag screw may result in cut out in Gamma Nails. But we still need to investigate more cases to further prove it.

There are still some drawbacks in our research. Firstly, the sample size is not large enough but we suppose that it is sufficient to illustrate our point. Secondly, in addition to our clinical research and we suggest to perform another biomechanical experiment to investigate the differences between the two set-screw fixing formations of the Gamma Nail.

It is evident that the Gamma Nail is a useful and effective system to secure the stable intertrochanteric fractures and equal to its counterpart, the DHS. The hip function one year postoperatively is satisfactory, no matter if the sliding function of the set-screw was used or not. Furthermore, the bone healing rate was similarly high in both groups. Considering the complications of lower limb shortening, the sliding of the lag screw doesn’t show any advantages in Gamma nail. As a matter of fact, when using the Gamma Nail, the lower limb discrepancy can be found when the lag screw has not been tightened with the set screw and thus a sliding ability. Moreover, the more comminution the fracture has, the lower limb discrepancy will be observed for A1 and A2 AO-OTA intertrochanteric fractures.

As a result, we can conclude that the sliding distance is minimal in Gamma nails and it is related to the comminuted extent of the intertrochanteric area in A1 and A2 AO-OTA intertrochanteric fractures. For treating these kinds of fractures, the sliding of the lag screw of an Gamma nail does not improve any clinical results and in certain cases, such as highly comminuted A1 and A2 fractures, can therefore even benefit from a locked lag screw by tightening the set-screw.

## Competing interest

The authors confirm that there are no known conflicts of interest associated with this publication and there has been no significant financial support for this work that could have influenced its outcome.

## Authors’ contributions

YZ carried out the study design, data collection, analysis, and manuscript writing. MS was involved in the data collection, manuscript revision and editing. CFL and CQZ contributed to the data collection and finalization of the manuscript. All the authors read and approved the final of the manuscript.
